# A Balanced Risk-Benefit Analysis to Determine Human Risks Associated with Pyrrolizidine Alkaloids (PA)—The Case of Herbal Medicinal Products Containing St. John’s Wort Extracts (SJW)

**DOI:** 10.3390/nu10070804

**Published:** 2018-06-22

**Authors:** Michael Habs, Karin Binder, Stefan Krauss, Karolina Müller, Brigitte Ernst, Luzia Valentini, Michael Koller

**Affiliations:** 1 Faculty of Medicine, LMU—University of Munich, 80336 Munich, Germany; info@michael-habs.de; 2 Didactics of Mathematics, University of Regensburg, 93053 Regensburg, Germany; karin.binder@mathematik.uni-regensburg.de (K.B.); stefan.krauss@mathematik.uni-regensburg.de (S.K.); 3 Center for Clinical Studies, University Hospital Regensburg, 93053 Regensburg, Germany; karolina.mueller@ukr.de; 4 General Medicine Unit, University Hospital Regensburg, 93053 Regensburg, Germany; dr.b.ernst@t-online.de; 5 Institute of Evidence-Based Dietetics, University of Applied Sciences Neubrandenburg, 17033 Neubrandenburg, Germany; valentini@hs-nb.de

**Keywords:** pyrrolizidine alkaloids (PA), risk-benefit analysis, St. John’s wort extract (SJW)

## Abstract

Objectives: Pyrrolizidine alkaloids (PA) exist ubiquitously in our environment. More than 6000 plants, about 3% of the world’s flowering plants, are known to synthesize PA. As a consequence, many herbal ingredients, including St. John’s wort (SJW), are contaminated with PA that can possess acute and subchronic toxic effects as well as mutagenic and genotoxic properties. Therefore, the possible benefits of SJW as an herbal remedy against depression need to be weighed against the possible risks of unwanted PA intake. Methods: We searched the literature regarding the current knowledge on PA and evaluated the evidence on the antidepressant effects of quantified SJW extract based on a Cochrane Review and the current practice guidelines on depression. Risks are depicted in form of a risk ladder and benefits in form of an icon array. Results: Evidence from clinical studies indicates that quantified SJW extract is an effective treatment option for mild to moderate depression with fewer side effects than conventional antidepressants. Health statistics from different countries do not quantify cases of death caused by PA intake. However, deaths due to suicide, often triggered by depression, are common (11 in 1000 in Germany in 2015) and rank between fatalities due to liver diseases (16 in 1000) and household accidents (10 in 1000). Conclusions: Quantified SJW extract is a safe and effective treatment option, and its potential of treating depression outweighs the (hypothetical) risk of unwanted PA intake.

## 1. Introduction

Pyrrolizidine alkaloids (PA) that exist ubiquitously in our environment possess mutagenic and genotoxic properties and can induce acute and subchronic toxic effects [1]. More than 6000 plants (about 3% of the world’s flowering plants) are known to synthesize more than 660 different PA [1,2,3,4]. Thus, PA can contaminate many biological products, such as foodstuff, beverages (milk, teas, and herbal infusions), and herbal remedies [4,5,6].

The case of St. John’s wort (SJW, hypericum perforatum) is of particular importance because it is a popular therapeutic option for one of the major diseases of modernity, namely depression [7]. The present paper puts PA-related risks resulting from temporary use of SJW in perspective with more general health and everyday life risks.

## 2. Pharmacological Effects of St. John’s Wort (SJW) in the Treatment of Depression: Clinical Studies, Side Effects, and Guidelines

### 2.1. Definitions

In Europe, the use of SJW as a medical remedy against depression has been documented since the first century AD [8]. However, in the context of this review SJW is considered for the group of herbal medicinal products with well-established use as defined in the Community herbal monograph on *Hypericum perforatum* L., herba (well established medicinal use): Herbal preparations with a dry extract (DER 3–7:1), extraction solvent methanol (80% *v*/*v*) or a dry extract (DER 3–6:1), extraction solvent ethanol (80% *v*/*v*) or a dry extract (DER 2.5–8:1), extraction solvent ethanol (50–68% *v*/*v*) [9]. Therefore, we will use the terms “SJW” and “quantified SJW extract” synonymously in this review.

The diagnoses of a depressive/major depressive episode overlap considerably [10]. This terminological indistinctiveness is also seen in clinical studies on depression. The title of the Cochrane review that we are going to describe below (see Section 5.2) reads “St. John’s wort for major depression” [11]. Later on in the review it is specified that “the severity of depression was described as mild to moderate in 19 trials, and as moderate to severe in 9 trials” ([11], page 7).

### 2.2. European and Chinese Studies

SJW is considered a medicinal product in Europe and China but a finished dietary supplement product in the United States (U.S.). Therefore, we present the following studies according to this geographical distinction.

In 2010, the large body of evidence resulting from the controlled use of standardized SJW in Europe has resulted in a “Community herbal monograph on hypericum perforatum” [12]. In this monograph, two distinct usages of SJW in solid dosage form are summarized for oral application, one for the treatment of mild to moderate depressive episodes (according to ICD 10) and the other for the short-term treatment of symptoms of mild depressive disorders. SJW is a prominent example of the successful translation of experience-based medicinal knowledge into a modern concept of evidence-based phytopharmacy.

Preclinical and clinical studies on SJW have been predominantly conducted in German-speaking countries with herbal medicinal products containing standardized extracts [13]. In vivo and in vitro studies have consistently shown that SJW acts on dopaminergic, serotonergic, and noradrenergic systems with complex effects. These activities are mediated by different types of constituents with hyperforin and probably hypericin as the main active ingredients. Animal pharmacology showed SJW to be active in behavioral models of depression, including learned helplessness and forced swimming tests. The experimental pharmacology of SJW has been investigated in numerous studies [14,15]. Consequently, the main human indication for SJW has been established in patients with mood disorders.

A Cochrane Review [11] has suggested that SJW is better than placebo and as effective as conventional standard antidepressants for mild to moderate depression but with fewer side effects. In the same vein, a more recent review [16] including 6993 patients from 35 studies has shown that SJW given as a monotherapy for mild to moderate depression is superior to placebo in improving symptoms and not significantly different from the conventional verum control drugs. Adverse events of SJW were comparable to those of placebo and fewer than those reported for standard antidepressant medication. A meta-analysis comparing SJW with selective serotonin reuptake inhibitors (SSRIs) in adults comprised 27 studies with 3126 patients, including 1177 patients from the People’s Republic of China [17]. The authors concluded that the efficacy of SJW is comparable to that of SSRIs in the treatment of depression, especially in the case of mild to moderate depression, but with significantly lower rates of adverse events and withdrawals.

### 2.3. U.S.-American Studies

In the U.S., SJW has not been approved as a drug by the Food and Drug Administration (FDA) but is commonly used as a finished dietary supplement product [18]. Little information is available on the pharmaceutical quality of these dietary supplements.

Recommendations by the National Center for Complementary and Integrative Health (NCCIH) focus on two clinical studies sponsored by the U.S. government [18]. Both studies were three-arm studies, one using sertraline and the other citalopram as active comparators. In both studies, the standard verum treatment and the SJW preparation failed to demonstrate significant superiority over placebo. This result may be due to the low sensitivity of the main outcome measures Hamilton Depression Scale (HAM-D total) and Clinical Global Impression Score (CGI) as well as due to a high placebo response [19,20]. In consequence, NCCIH statements do not support the use of SJW containing supplements for persons with depressed mood and underline possible risks associated with self-medication with SJW supplements.

### 2.4. Side Effects of SJW

Side effects of SJW become particularly apparent in combination with other substances, such as cyclosporine, tacrolimus, amprenavir, indinavir, and other protease inhibitors, irinotecan and warfarin. To avoid interactions with substances metabolized by CYPP3A4, CYP2C9, CYP 2C19, or P-glycoprotein, special care is recommended in the case of concomitant use, as specified in the European Community Monograph and matched by national professional product information [12,21,22,23,24,25]. Because oral contraceptives are among those drugs, women taking the pill are advised to use additional contraceptive measures. In the absence of sufficient data, use in patients under 18 years and during pregnancy and lactation is not recommended [12]. Furthermore, intense UV-exposure should be avoided. Hypericin and related naphthodianthrones cause phototoxicity due to UV light sensitivity, which has been shown in experiments (in vitro and in vivo) and in humans [26].

Therapy with SJW should follow a medical rational [4], should be administered as a monotherapy, and is not suitable for polypharmacy. SJW products are not sufficiently investigated to be recommended for combination therapy with psychotropic drugs or medications for other indications. The interaction potential limits SJW use in combination protocols [24,27].

### 2.5. Guidelines and Cross-Cultural Differences

The indications given in EMA/HMPC Monographs reflect the consensus of the representatives of the national regulatory bodies represented in this committee. The mission of this committee is to support the harmonization of the European Market by providing unified opinions. On the other hand, in 2009 the German Federal Institute for Drugs and Medicinal Products (BfArM) approved SJW herbal medicinal products with the indication moderate depression mandating a prescription-only status. Therefore, it may be superior to rely on the published original clinical data and meta-analyses when assessing the efficacy and safety of herbal medicinal products and not limit the focus towards the (non-binding) HMPC statements.

The current German national healthcare guidelines for unipolar depression are based on a consensus of representatives from all major medical societies and professional associations in Germany, self-help groups, and associations of family members and relatives [28]. Antidepressants should generally not be used for the initial treatment of mild episodes but may be indicated based on a patient’s wishes and preferences, a patient’s positive experiences in the past, continued symptoms after alternative interventions, and if recurrent episodes of depression are known from a patient’s history. Drug treatment is recommended for acute moderate depressive episodes. SJW is frequently prescribed due to the perceived better side effect profile. However, since the constituents of the SJW extract differ among individual manufacturers, the effectiveness cannot be extrapolated from one extract to another. Thus, only medicinal products with clinically documented efficacy should be used [29]. Furthermore, the specific side effects of SJW and interactions with concomitant medication have to be considered (see Section 2.4).

The guideline on the treatment of adult patients with major depressive disorders issued by the American College of Physicians refers to the same studies on SJW as the German guideline. However, the guideline does not give a recommendation in favor of SJW because “St. John’s wort is not currently regulated by the U.S. Food and Drug Administration, and there is no current standard in place about the contents and potency of the medication. Therefore, patients in the United States may not be able to get a quality-controlled medication or reliably obtain preparations with similar effectiveness as those used in the included studies” [30].

It becomes evident that there is a cross-cultural element to the use and recommendation of SJW. In Europe, SJW has been in use for decades, and pharmacological standards for the production of SJW-based medicinal products have been established. This is not the case in the U.S., with no FDA regulation on the making and use of SJW products being in effect.

## 3. SJW Contamination with PA

**Figure 1:**
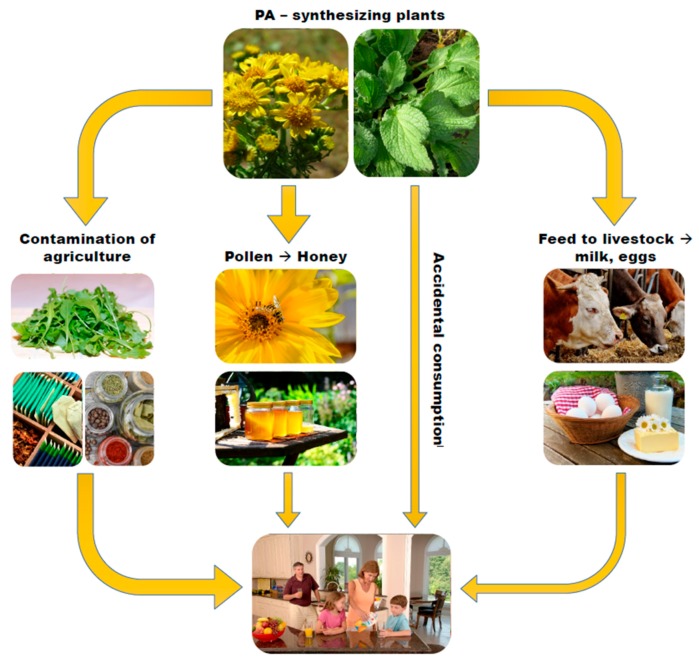
Four ways of how PA can enter the human food chain.

Generally, there are four ways of how PA can enter the human food chain (Figure 1):

1. Contamination with PA-synthesizing weed in agricultural cultivations [1,2]: PA contamination of tea and herbal infusions is frequent and attributed to contamination of the raw materials that are harvested along with PA-forming weeds [4].
2. Contamination of pollen of plants and resulting bee products such as honey: The PA content of honey has been shown to be directly proportional to the amount of PA pollen in honey, and the transfer of PA from pollen to honey occurs rather quickly [31].
3. Direct, accidental, or excessive consumption of PA-synthesizing plants: In Germany, borage (Borago officinalis) is a prominent example. This herb is one of the characteristic ingredients of “Frankfurt Green Sauce”, a traditional dish in the State of Hesse [32,33].
4. PA-contamination of feed given to livestock: Such contamination may result in low level PA contamination in the foods produced by the animals such as milk and eggs.

From the available analytical data, it can be summarized that the PA burden for the general population results primarily from the consumption of tea and herbal infusions [2,34].

Nevertheless, SJW may be contaminated with PA. In Europe, the European Medicines Agency (EMA) and the national regulatory bodies cooperate with herbal pharmaceutical companies and associations to keep PA contamination in herbal medicinal products as low as possible. At present, patient exposure to PA from herbal medicinal products should not exceed a daily intake of 1 μg. For the future, EMA strives for a limit value of 0.35 μg per day [35]. In 2016, SJW tablets were recalled from the UK market [36] because of higher than recommended levels of PA. A recent survey from German supermarkets, drugstores, and pharmacies identified that SJW products frequently contain PA concentrations above the recommended values of the European and German regulatory bodies. Only about 30% of the drawn samples were of accurate quality [37].

This low percentage highlights the importance of regulatory controls. In Europe, the legal constraints for manufacturers of herbal drugs are generally stricter than for foodstuff due to different legal systems: in principle, food is assumed to be safe, whereas drugs must show a positive risk-benefit ratio before market approval, and post-marketing surveillance is mandatory. The rigid regulatory standards of medicinal products compared to food regulations help to monitor the burden from PA contamination in this product category [35,38]. In 2016, a detailed “Code of practice to prevent and reduce PA contaminations of medicinal products of plant origins” has been published by German manufacturer associations [39].

SJW herbal medicinal products are used for the symptomatic treatment of mood disorders and depression; hence, doses and length of treatment vary. The length of controlled clinical trials ranges between 4 to 12 weeks [17]. In general, treatment periods with SJW are in the order of magnitude of months but not years. When using SJW, the exposure time of possible PA contamination of this herbal product is clearly less than that of people who regularly drink tea or herbal infusions that are frequently contaminated with PA. As a single source, PA contamination of herbal medicinal products with SJW as the active ingredient is irrelevant. The many possible origins of PA contamination [40] underline the necessity to substitute the pure risk analysis with a specific risk-benefit analysis.

## 4. PA Toxicity: Detectability, Metabolism, and Serious Health Consequences

The progress in recent years in PA analytics (HPLC and tandem mass-spectrometry) has resulted in the ability to detect PA at trace levels in nearly every part of the human environment [41,42]. Harmonization of methods to achieve comparable results in different chemical laboratories still needs to advance further [2,3,43,44,45,46]. Better detection methods should not be confused with an overall increase in PA occurrence [35], for which there is no evidence [2,3,6,47].

Toxicity requires metabolic activation involving cytochromes P-450 and flavin-containing monooxygenases [5]. Toxification and detoxification pathways are interrelated and vary greatly within and among species, the two sexes, individuals, and over time [48,49,50].

We have recently reviewed the PA experimental toxicology and related findings in humans [4]. Hepatotoxicity is the main effect after PA exposure. Pulmonary toxicity is sometimes seen after exposure to hepatotoxic PA. The same structural requirements and the same metabolites are necessary for liver and lung toxicity. The more stable the dehydropyrrolizidine metabolites, the easier can they be transported away from the liver and cause toxic effects in other organs.

Consumption of high doses of PA may result in veno-occlusive liver disease, the occlusion of central venules [51,52,53]. Case reports associated with PA-contaminated grain have been reported in Pakistan, India, and Afghanistan. This type of massive grain contamination with PA-synthesizing plants is unlikely to occur in modern professional agricultural systems [54].

PA-contaminated food has never been studied for carcinogenicity in long-term animal studies. Any carcinogenic effect due to PA exposure would be expected to follow long-term, low-dose exposure. Many human environmental carcinogens manifest only after long latency periods, thus early in life exposure, and life-time burden seems more important in adulthood than short-term exposure [55].

However, hepatic vein occlusion, mutagenicity, genotoxicity, and carcinogenicity of PA and PA-containing plant extracts have been studied since the mid-1970s [56] and evaluated by the International Agency for Research on Cancer (IARC) resulting in categorization as Group 2B (possibly carcinogenic to humans) or Group 3 (not classifiable as to its carcinogenicity to humans). Lasiocarpine, monocrotaline, and riddelliine are Group 2B carcinogens [57,58,59]. Mechanistic studies have shown dehydropyrrolizidine to form DNA adducts that could well be the causal mode of action resulting in mutagenicity and cancerogenicity [3,60].

In rodents, the signal tumor of long-term PA poisoning is hepatic hemangiosarcoma [61,62]. Additionally, hepatocellular adenoma and carcinoma have been reported [63]. If rare tumor types found in humans correspond to tumors seen in animals exposed to the same suspected carcinogenic agent, such findings are accepted as a strong empirical argument for causality. About 50% of the products identified by IARC as carcinogenic to humans show comparable organotropism and tumor pathology in animals. Human carcinogens associated with liver carcinoma and hemangiosarcoma include Alpha-emitters, vinyl chloride, and arsenic. Corresponding tumors can be induced in animals [4,64,65].

Detoxification capacity by glutathione conjugation in sinusoidal cells is less than in hepatocytes due to lower glutathione content, leading to higher intercellular exposure to toxic free radicals. An overload of the intracellular detoxification systems prompts the hepatotoxicity of monocrotaline. This observation suggests a threshold dose for this type of toxicity.

The incidence of human hepatic hemangiosarcoma is very low [66,67]. No evidence exists for an increase in this tumor type over time, and no epidemiological data show any association between chronic low-dose PA exposure and human disorders [2,35,43]. Angiosarcoma derives from endothelial cells of blood vessels or lymphatic vessels. Angiosarcoma is estimated to account for 2-3% of all types of soft tissue sarcoma, and primary hepatic angiosarcoma accounts for <5% of all types of angiosarcoma. Based on epidemiological data from Norway, Sweden, the UK, and the U.S., a review yielded an annual incidence of 0.5 to 2.5 cases per 10,000,000 individuals. Among those cases, 20–25% can be linked to known etiologic factors, for instance vinyl chloride monomers [68,69,70,71] that are used to produce polyvinyl chloride, a widely used plastic. The IARC has classified polyvinyl chloride as a carcinogen with sufficient evidence of inducing hemangiosarcoma of the liver in exposed workers. There is also strong evidence that polyvinyl chloride induces hepatocellular carcinoma as an occupational disease [64]. No evidence has been produced to link environmental exposure to vinyl chloride monomer with human angiosarcoma of the liver [67]. The goal of effective risk assessment is to prevent morbidity and mortality in humans. Therefore, risk assessment is based on instances that are observable and quantifiable. Certainly, we cannot exclude unknown facts, which leaves room to speculate about the theoretical risk of human tumors of unknown organotropism and their association with low-dose PA exposure in the case of unknown, but specific individual disposition. However, risk assessment is considered an evidence-based discipline that should provide state-of-the-art information about specific quantified health issues [4,40,43,72,73,74,75].

Taking the extensive follow-up reports on human PA exposure into account, Prakash et al. concluded that the human risk of veno-occlusive disease and childhood cirrhosis are established but also that PA are not carcinogenic to humans [76].

From a scientific standpoint, however, we must adhere to the principle “absence of evidence is not evidence of absence” [4,77]. Thus, just because we could not find any PA-induced tumors in humans, the counter argument that a PA-tumor link does not exist is not confirmed. Nevertheless, what we can infer from the current evidence is that the risk of human angiosarcoma due to PA contamination must be so small that it cannot be quantified through health statistics (see Table 1). In contrast, other well-known health or everyday life risks (e.g., suicide and household accidents) are preventable but still exist and are rarely addressed in public debates.

Regarding primary liver cancer, we calculated the total number of liver cancer minus the number of cancers attributable to risks from known sources for the U.S. [4]. The conservative estimate of annual new cases not due to hepatitis is below 5000. These <5000 cases must include all cases from known risk factors, excluding hepatitis B and C. Major well-established risk factors are obesity, metabolic syndrome and diabetes, hemochromatosis, alcohol consumption, and aflatoxin in warmer and tropical countries [78].

Taking the conceptions on the mode of action for chronic PA toxicity together with the experimental findings in long-term animal studies, we should expect hemangiosarcoma of the liver and hepatocellular carcinoma to be the most likely results after long-term and low-dose PA exposure in humans [4]. Nevertheless, given the low prevalence of hemangiosarcoma, the causal contribution of PA contamination must be very small compared to the impact of the sum of primary liver cancer attributable to all well-recognized sources.

## 5. Balanced Risk-Benefit and Risk Communication

In the light of the potentially harmful effects of PA and the clinical data available on SJW that so far failed to provide evidence for a human link between PA and SJW, we try to put benefits and possible risks into a broader perspective. More specifically, we will quantify and summarize the benefits and potential risks of SJW intake as an herbal medication for mild and moderate depression, using two established risk communication tools, namely risk ladder and icon array.

### 5.1. Rationale for and Methods of Compiling the Risk Ladder

The risk ladder is a standard method to show risks in perspective by ranking common causes of death in a descending order. Our source data are original counts and numbers as reported in statistical reports and data bases that are standardized by applying a common anchor. Thereby, the relative size of diverse everyday risks and health risks becomes apparent. Such risks ladders provide astonishing insights, for instance that suicides are more frequent than traffic accidents. One major reason for such surprise experiences is the availability heuristic, which renders cognitive contents that are highly publicized and thus readily available to be associated with higher levels of risks [79]. This preconception often breaks down when one looks at the real evidence.

For the risk ladder, we selected major illnesses responsible for major health costs, such as cardiovascular disease, cancer, diabetes, diseases that may be caused or aggravated by toxic substances (including PA), such as liver disease or liver cancer, and common everyday fatalities, such as traffic or household accidents. In order to allow for a representative comparison of relative risks over time and across countries, we consulted different sources: official death statistics issued by the German Statistical Federal Agency, the Swiss Statistical Federal Agency, the Center for Disease Control (CDC), and the Global Burden of Disease (GBD) Collaboration (all respective web links can be found below Table 1).

The German cause of death statistics is an annual census [80]. The data are based on the official death certificates that are transferred from the health authorities to the statistical offices of the federal states. In the case of missing or implausible information, the health authorities clarify the discrepancies with the doctors. Cause of death is coded according to the ICD 10, ensuring the international comparability of the data. Inaccuracies may arise from the information provided by physicians and the subjective evaluation of the causes of death in the statistical offices of the federal states. In order to minimize such errors, data are continuously random-checked. The findings are used for the annual training of specialist staff in the statistical regional offices.

Likewise, the Swiss cause of death statistics are based on a census [81]. The percentage of missing information on the cause of death is estimated to be 2%. The standardization of data collection and the use of the ICD code guarantee good international comparability.

CDC.gov is the official Web site of the CDC, which is the leading U.S. public health institute under the Department of Health and Human Services (headquartered in Atlanta, Georgia). CDC.gov is a public domain website providing direct access to important health and safety topics, scientific articles, data, statistics, tools, and resources.

*Our World in Data* is an online publication providing an array of statistics on changing living conditions that is produced and maintained by the University of Oxford as a public good. Data stem from different sources, namely specialized institutes, research articles, international institutions, and international statistics. A key is the GBD collaboration that employs a standardized approach to the attribution of deaths to specific causes based on limited data [82]. The GBD mortality cause assessment is in accordance with and strongly tied to categories as defined within the ICD.

### 5.2. Methods of Compiling the Icon Array

**Figure 2:**
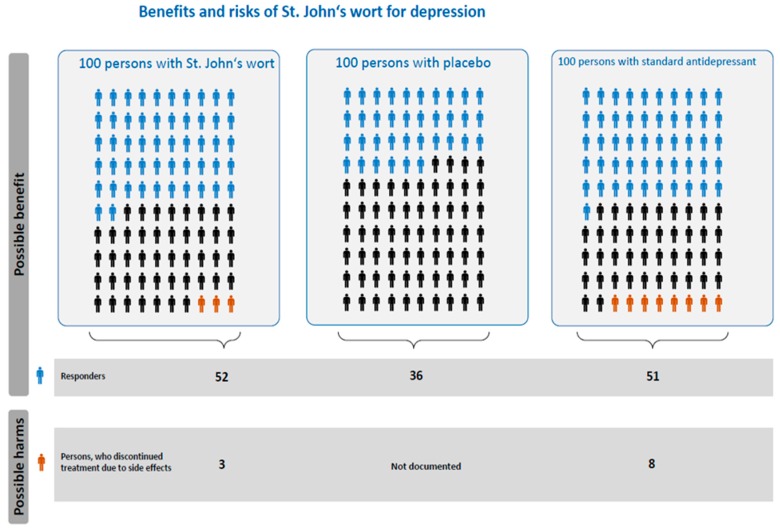
Icon array-graphic depiction of the Linde et al. (2008) Cochrane Review [11].

Icon arrays consist of a matrix of icons that represent a population at risk and simultaneously display the number of events (e.g., responders) and non-events (e.g., non-responders) [72,83]. Icon arrays have the property to visualize at one glance the part-whole relationship and can also be used to depict features other than efficacy (e.g., responders vs. non-responders), for instance side effects (e.g., suffering from a special side effect vs. not suffering from a special side effect). Furthermore, to compare benefits and risks of different kinds of treatments (e.g., standard antidepressant vs. SJW vs. placebo), three icon arrays—one for each treatment—can be depicted side by side. In the present icon array (Figure 2), the summary of chances and risks are also depicted numerically on the bottom of the icon array, following the idea of icon boxes of the Harding Center for Risk Communication [84].

The present icon array is based on the Cochrane Review by Linde et al. [11], which is the most comprehensive and most prestigious review on SJW. The Cochrane Review was based on 29 RCTs. A variety of hypericum preparations were investigated, and in most trials the extract doses ranged between 500 to 1200 mg. Standard antidepressants were used as active comparators. The Cochrane Review presents absolute numbers (not only relative risk reductions), which are necessary to generate icon arrays. In contrast, more recent reviews published in 2016 presented relative risks and were therefore not included in the icon array. It is important to note, however, that the 2016 reviews echo the conclusion drawn from the Cochrane publication [16,17]. The review conducted by the Chinese author group was based on 27 RCT that compared standardized SJW extracts to SSRIs. The RCTs were conducted in Brazil, Canada, Denmark, Germany, the People’s Republic of China, and the USA. The quantified SJW extracts included Calmigen, Iperisan, LI-160, LoHyp-57, STW3, WS5570, and Ze117 and were typically administered in a dosage of 900mg/d for 6 to 12 weeks [17]. The same is true for the analysis by Apaydin et al. [16].

Thus, the available body of knowledge on clinical data with regard to SJW has not changed over the past decade.

### 5.3. Interpretation

Risk comparison within a risk ladder improves risk understanding [72,73,74,85]. The risk ladder shown in Table 1 compares the death rates of major health risks as well of everyday life risks that should help to put the possible risk of PA contamination from SJW medicinal products into perspective. Comparing figures from different sources and for different cohorts show a consistent order of magnitude. Deaths from cardiovascular diseases and cancer by far dominate all other life risks. Fatal household accidents and traffic accidents in developed countries are outnumbered by the risk from suicide. Neuropsychiatric diseases are the number one risk factor of suicidal death. Depression accounts for up to 60% of suicides [86,87,88]. Evidence indicates benefits of antidepressant medication in patients who experience suicidal thoughts and are prone to suicidal behavior [89]. A recent meta-analysis of the efficacy and acceptability of 21 antidepressants published in the Lancet [90] showed all individual medications to be superior to placebo when comparing an 8-week period after the onset of treatment. The Lancet review did not mention SJW, probably because SJW is a non-prescription herbal remedy in the UK. The cross-cultural component in assessing SJW has already been noted earlier. However, in controlled clinical trials, quantified SJW extracts have shown to be superior to placebo and as effective as synthetic antidepressants, both classic tryciclics and newer SSRIs [12,27]. Nevertheless, the safety profile of SJW as indicated by dropout rates from clinical studies and unwanted drug effects is in the placebo range. Thus, lower withdrawal from studies due to adverse events by SJW is an advantage in the management of major depressive disorders [91].

Based on the current combined evidence on efficacy, side-effect profile, and adherence, the use of quantified SJW extract as monotherapy in patients with mild to moderate depression is a rational choice.

## 6. Points to Consider and Conclusions

PA contamination in the human environment has been recognized for decades. Recently, interest has risen due to improved analytical methods and a steadily increasing number of analytical reference standards for individual PA. No evidence exists for an increase in PA uptake in the general population in the past. Main sources for PA contaminants in humans are food, beverages, supplements, and herbal remedies. For the general population, beverages and foodstuff derived from natural sources are more important than supplements and herbal drugs. A major impact results from habitual drinking of tea and herbal infusions. In a recent project, we have performed a risk-benefit analysis to balance possible human risks associated with PA contamination with the benefits resulting from drinking tea and herbal infusions. We concluded that the benefits of drinking tea and herbal infusions clearly outweigh the health risk of possible PA contamination that was judged to be negligible [4].

In the present paper, we extend our risk-benefit analysis to herbal medicinal products, choosing SJW as a prominent and widely used agent. Patients taking contaminated herbal medicinal products (or supplements resulting from natural sources) add a treatment-specific possible PA burden to their background exposure for a limited time. For herbal medical products used according to the Community Monograph on SJW [12], clinical effectiveness comparable to that of conventional SSRI has been established; adverse events were comparable to placebo, and side effects were fewer than seen with standard antidepressant medication. This clearly makes SJW herbal medicinal products suitable as a first line medical treatment option for patients with mild to moderate depression, particularly for patients who favor phytotherapy over conventional medicinal products. Upper limits for PA contamination for herbal drugs exist and the number of depressive patients on long-term SJW treatment is limited. Thus, the additional PA exposure during drug treatment is expected to stay well within the boundaries given for the general population by differences in interindividual life style and nutrition [2,5,34,35].

This conciliatory conclusion, however, is no excuse for manufacturers to discontinue their efforts to continuously secure good product quality and be transparent in their measures to achieve this goal, since a recent analysis has clearly shown room for improvement [79].

## Figures and Tables

**Table 1:** Risk ladder.

Death Cases	2015 * Germany	2008 * Germany	2016 ** World	2014 *** Switzerland	2014 **** United States
Absolute number of deaths	925,200	844,439	54,700,000	63,938	2,626,418
Standardized at 1000 cases					
Cardiovascular disease	385	420	323	328	234
Cancer	250	257	163	262.3	225
Diabetes mellitus	26	26	58.3	19	29
Liver diseases	16	18	23	14.4 *****	15
Suicide	10.9	11.1	14.9	16 ^1^	16
Household accidents	10	8.1	23 ^2^	10.3	6.9
Liver cancer	8.5	8.3	12.3 *****	9.4	9.4
Traffic accidents	3.8	5.5	24.5	3.6	12.8
Liver cancer (unexplained cause)	1.7	0.9 *****	3.5 *****	1.8 *****	2.1 *****
PA-related death ^1^	?	?	?	?	?

^1^: Suicide and assisted suicide; ^2^: (2015); * Source: Federal Statistical Office, Germany, Annual Report 2015 and 2008; ** Source: www.ourworldindata.org (Global Burden of Disease (GBD); *** Sources: bfu—Swiss Council for Accident Prevention. STATUS 2017; https://www.bfs.admin.ch/bfs/de/home/statistiken/gesundheit/gesundheitszustand/sterblichkeit-todesursachen/spezifische.html#par_text; https://www.nzz.ch/schweiz/aktuelle-themen/krebskranke-mehr-patienten-und-tieferes-sterberisiko-ld.9031. *** Source: https://www.nzz.ch/schweiz/aktuelle-themen/krebskranke-mehr-patienten-und-tieferes-sterberisiko-ld.9031. **** Sources: https://www.cdc.gov; https://www.asecurelife.com. ***** Source: https://vizhub.healthdata.org/cod/. ? unknown, not listed in any death statistics.
